# Quantifying uncertainty of tuberculosis drug susceptibility range from single-microplate test

**DOI:** 10.1016/j.mex.2026.103797

**Published:** 2026-01-14

**Authors:** Eugene B. Postnikov, Anastasia I. Lavrova

**Affiliations:** a Department of Theoretical Physics, Kursk State University, Radishcheva st., 33, Kursk, 305000, Russia; b Sophya Kovalevskaya North-West Mathematical Research Center Immanuel Kant Baltic Federal University, 14 A. Nevskogo str., Kaliningrad, 236016, Russia; c Saint-Petersburg State Research Institute of Phthisiopulmonology, 2-4 Ligovsky av., Saint-Petersburg, 191036, Russia

**Keywords:** Uncertainty quantification, Small sample statistics, *M. tuberculosis*, Pharmacokinetics

## Abstract

The registration of fluorescence intensity converted from resazurin in the course of the respective microtiter assay, as a response to the activity of antibacterial drugs, provides a basis for the quantitative characterisation of their activity. At the same time, this assay operates with relatively small samples, and the studied biochemical process exhibits variability. In this work, we address the question of reporting the minimal inhibitory concentration arguing in favour of its range rather than a single value. To achieve this goal, we propose a method for the combinatorial enhancement of single microplate-based data, followed by non-parametric statistical processing. The approach is illustrated by the case study of ten first- and second-line anti-tuberculosis drugs acting on the standard laboratory strain H37Rv of *Mycobacterium tuberculosis*.

## Highlights

• A numerical uncertainty range quantification from single-plate experiments.
• Overcome possible overfitting with the four-parametric Hill’s function.
• Processed dataset for first- and second-line antimycobacterial drugs.

## Graphical abstract

**Figure:**
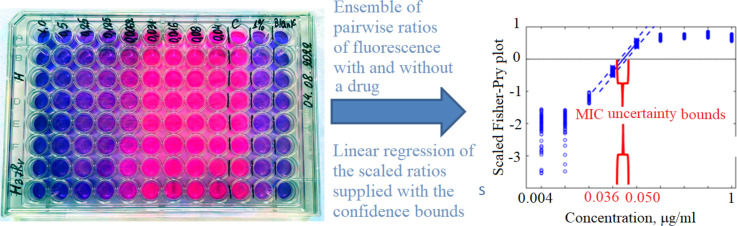

**Table:** 

**Specification Table**	
Subject area	Pharmacology, Toxicology and Pharmaceutical Science
More specific subject area	Drug susceptibility testing
Name of your method	Single-plate uncertainty quantification
Name and reference of original method	Not applicable
Resource availability	https://github.com/postnicov/MICuncertainty

## Background

**Fig. 1:**
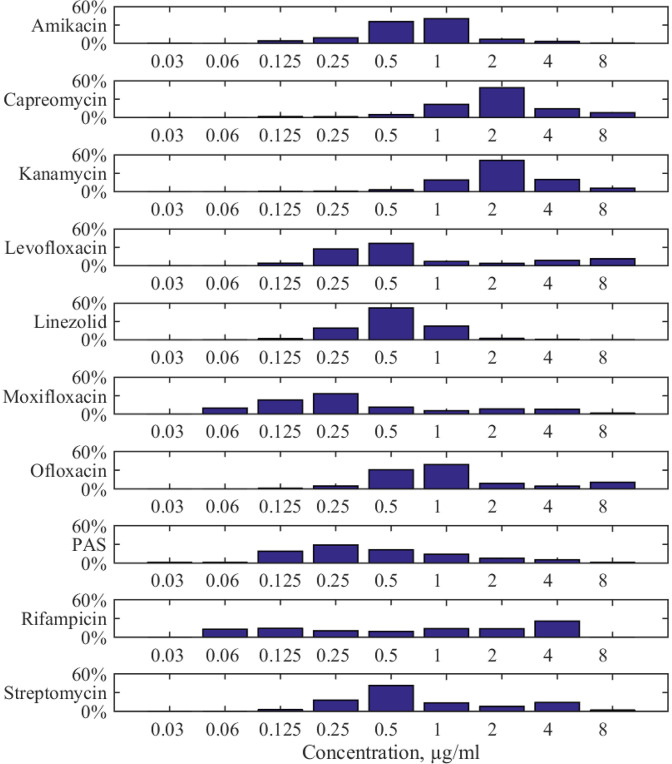
Examples of the distributed MIC values for ten conventional antimycobacterial drugs acting on the wildtype of *M. tuberculosis* according to the data made publicly available by the European Committee on Antimicrobial Susceptibility Testing (EUCAST, https://mic.eucast.org/, accessed 30.05.2025). The percentage for the data reported within the range (0.03−8)μg/ml is shown.

Among specific areas, where uncertainty quantification can be crucial, is the field of biomedical science since operations with living objects lead to data with high scattering and quantitative arguments are strictly needed for developing healthcare system [1]. In particular, such characterization is required when developing new efficient drug candidates [2], [3] and testing possible evolving resistivity to existing ones [4], [5]. In general, it is an extremely wide area; thus, in this work we will focus on a particular case of antimycobacterial drugs’ action. Tuberculosis is not only still one of the worldwide treats but *Mycobacterium tuberculosis* has variable sensitivity on the intra-strain level and actively develops its drug resistance. As a result, the reported data on the minimal inhibitory concentration (MIC) even for conventional first- and second-line drugs are distributed in a wide range, see Fig. 1. The World Health Organization has attracted attention to the necessity of consideration of the respective distributions rather than single values [6].

Among widespread routine methods for determining the minimal inhibitory concentration (MIC) is the employing the Resazurin Microtiter Assay (REMA), which is based on the conversion of blue non-fluorescent resazurin (also known as Alamar Blur) to pink fluorescent resorufin [7], [8]. In addition to the simple quantitative determination based on the visual colour change of the indicator [9], the conventional quantitative definition of the MIC is based on the fluorometric mode of study operating either with the signal’s reduction by 90% relative to untreated cultures [7], [10] or with the regression of the dose-response curve [11]. For this purpose, the standard way of regression and interpretation of such kind of pharmacological data [12], [13] uses the dose-response data represented by the Hill equation

(1) $$f=f_{min}+\frac{f_{max}-f_{min}}{1+{\left(\frac{x}{IC_{50}}\right)}^{\alpha }},$$

which interpolates the change of the registered activity (in our case, it is the fluorescence intensity) *f* from *f*_max_ to *f_mix_* in response to the drug concentration *x* changing from x=0 to *x* → ∞. Here *IC*_50_ is the parameter equal to the concentration resulting in 50% inhibition, and *α* is the kinetic constant conventionally to considered it as an arbitrary positive phenomenological parameter characterizing the degree of sigmoidicity of the actual data fitting curve.

**Fig. 2:**
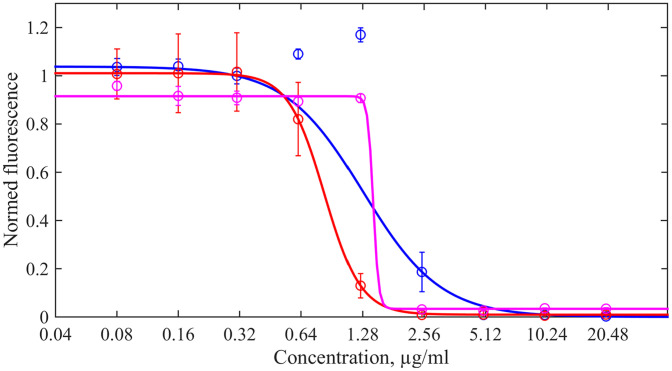
Examples of three repetitions of the REMA test with kanamycin acting on the strain H37Rv of *M. tuberculosis* shown by different colours. Markers correspond to median relative fluorescence; they are supplied with error bars defined by the scaled median absolute deviations over the data obtained from eight wells in the plate’s column. Solid curves are regressions with the Hill function given by Eq. (1).

Although Hill’s curves given by Eq. (1) reproduce the data point quite accurately for individual measurements, the parameters obtained from several repetitions can be drastically different as illustrated in Fig. 2 (more plots and numerical values of the Hill kinetic parameters can be found in Supplementary material). Although the determination coefficients are equal to $R^{2}=0.9992$, $R^{2}=0.9999$, $R^{2}=0.9986$ for the blue and red, and magenta curves, respectively, even the naked eye indicates that these kinetic curves correspond to significantly different kinetic parameters: α=2.3,4.9,33. Whence, the reporting the MIC and kinetic parameters from a unique set of averaged data can not be warranted. But in practice, one may encounter the need to interpret data obtained from a unique plate, say, when working with clinical strains. Therefore, the problem of building a method for uncertainty quantification under conditions of a limited number of experiments emerges.

### Experimental data

To illustrate the proposed method, we used datasets from the data archive of the Saint-Petersburg State Research Institute of Phthisiopulmonology (Saint-Petersburg, Russia) collected in 2016–2020. Each file consists of an Excel spreadsheet, which is the direct raw output of the FLUOstarOptima (BMG Labtech, Offenburg, Germany) plate reader (to see and access the original files, see ’Resource availability’ link in the Specifications table). The numerical values are blank-corrected fluorescence levels in each well of a standard 96-well microbiological plate. Eight columns contain results of the REMA test for binary dilutions of the acting drug; the data from the ninth column correspond to the fluorescence of resorufin produced by the unaffected mycobacterial culture. Ten examples of first- and second-line drugs acting on the standard laboratory strain H37Rv of *M. tuberculosis* were considered; the data consists of 2−4 microplate assays completed in separate time periods.

## Method details

As the first step, let us recall the conditions of the REMA method. For each concentration of a drug, there are eight measurements (i=1.8) of fluorescence {*F_ij_*} of the bacterial growth indicator medium (in eight wells in *j*th plate’s column). At the same time, there are also eight measurements {*F*_*i*0_} in the control column. We need to operate with relative data characterising the change in fluorescence. All 16 experimental wells are independent.

***The workflow’s step 1:** to form an enhanced statistical ensemble considering all possible 64 combinations* $f_{ij}=\left\{F_{ij}\right\}\{F_{i0}\}$ *instead of one point conventionally used by the averaging during the standard data preprocessing.*

It should be pointed out that such a procedure leads to the emergence of certain correlations between elements of the extended data set. However, this drawback is simultaneously compensated by an increase in the number of degrees of freedom. This increase can be regarded as a positive feature, as it allows for a more reliable interval characterisation of data measured by comparison with “reference units” exhibiting a certain degree of instability [14], [15].

This feature play a significant role in the considered case of the REMA test since it conventionally produces not more than 8 data points for the control and 8 data point for the test. Such a small sets does not allow ensemble enhancement by bootstraping since reduced subsamples with data replacement would be statistically underpowered. This limitation on the data amount restricts also the application of Bayesian methods based on empiric distribution functions.

Note also that in our case all elements of the set {*F*_*i*0_} correspond to the indicator fluorescence in absence of a drug, which is always significant. As a result, the ratio is free of statistical problems for ratios with significant amounts of small denominators leading to heavy tails of the respective distributions.

The second premise of the proposed approach is based on the fact of the existence of the principal ground truth for Hill’s kinetics described by Eq. (1). By definition, we operate with the relative fluorescence defined respectively to the untreated bacterial culture. This means that the true value for x=0 should be f=1. Respectively, if the inhibition of more than 90% exists, then the true value for *x* → ∞ is f=0. These two boundaries imply $f_{\max}=1$ and $f_{\min}=0$. Note that such true values have been used in the rare case of processing extremely extensive datasets, e.g., the work [11] when there was an opportunity to use a large number of repetitions. Other values of *f*_min_ and *f*_max_ are phenomenological adjustment parameters for the goal of fitting a particular limited data set that, as we demonstrated above, lead to undetermined scattered kinetic parameters.

In addition, Eq. (1) can be rewritten as the logistic curve

(2) $$f=f_{min}+\frac{f_{max}-f_{min}}{1+e^{\alpha (\ln\left(x\right)-\ln\left(IC_{50}\right))}}$$

when considered as a response to the logarithm of acting drug concentration. This representation is natural due to serial binary dilutions used in the microtiter assay. In the modern protocols for antimicrobial susceptibility testing, plotting the fluorescence data fitting curve as a function of the logarithm of concentration is included as one of the principal steps [16].

Using $f_{\min}=0$ and $f_{\max}=0$, the logistic form of HIll’s kinetic equation, Eq. (2) can be transformed to the so-called Fisher-Pry representation [17]

(3) $$\ln\left(\frac{1-f}{f}\right)=\alpha \ln\left(x\right)-\alpha \ln\left(IC_{50}\right),$$

which linearly depends on the logarithm of concentration.

The definition of the fluorescence-based MIC corresponds to f=0.1, i.e. (1−f)/f=9. Thus, from the computational point of view, it is better to use not the natural logarithm but

$$\ln\left(9\right)\log_{9}\left(\frac{1-f}{f}\right)=\alpha \ln\left(x\right)-\alpha \ln\left(IC_{50}\right)$$

or

(4) $$FP_{9}\equiv \frac{\ln\left(\frac{1-f}{f}\right)}{\ln(9)}-1=\frac{\alpha }{\ln(9)}\ln\left(x\right)-\frac{\alpha }{\ln(9)}\ln\left(IC_{50}\right)-1.$$

***The workflow’s step 2:** To substitute the points of the formed enhanced f_ij_ vs. the drug concentrations x in Eq. (*4*) preparing the pairs of logarithmed data (FP*9 *vs.* ln (*x*)*) for the subsequent linear regression.*

**Fig. 3:**
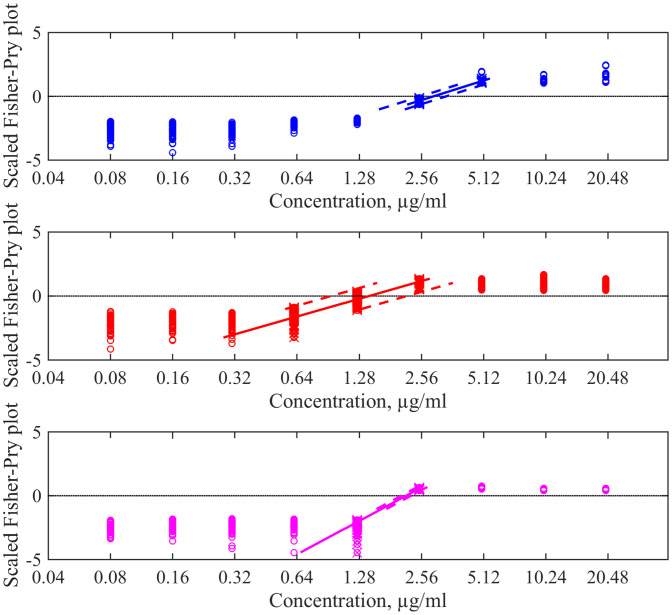
Examples of three repetitions of the REMA test data shown in Fig. 2 in the Fisher-Pry representation given by Eq. (4). Colours of data points and their regression lines around the zero crossing point in each subpanel are the same as in Fig. 2. Empty circles are elements of the enhanced experimental ensemble. The data surrounding the zero ordinate and used for the linear fit (solid straight lines) are highlighted by crosses. Dashed curves denote the prediction interval for the fits built around the zero crossing when its robust nonparametric detection is applied.

The advantage of Eq. (4) is that the logarithm of the minimal inhibitory concentration, ln (*x_MI_*), gives the zero crossing of the straight line stated by the right-hand side of Eq. (4). Examples of such representation for the same acting drug as in Fig. 2 are shown in Fig. 3 for each element of the extended ensemble (the unreliable points, for which *f* > 0, as well as points with f=0 are excluded).

Let us note that the right-hand side of Eq. (4) operates in fact with the differences in terms of the measured fluorescence levels

(5) $$\ln\left(\frac{1-f}{f}\right)=\ln\left(F_{i0}-F_{ij}\right)-\ln\left(F_{ij}\right).$$

This construction generalizes the well-known Hodges-Lehmann construction [18], which addresses the alignment of two independent sets of measurements at zero. The difference consists of the usage of logarithmic variables instead of original ones and existence some additional correctional bias in the extended ensemble. However, these features principally affect the tail parts of distribution and, therefore, less affect the region of zero crossing, which is our main area of interest.

Although strict statistical quantification for this particular case is a complicated analytical procedure and is outside the scope of the present work, one can refer to the work [19]. That study indicates that the undesirable effects are not crucial for a wide range of generalisations of the Hodges-Lehmann approach when robust non-parametric data processing is applied.

It is worth noting that very low and very high concentrations do not give points following a straight line. It is a known effect of noisy data in the logarithmic representation, discussed, e.g. in the original paper [17], where only the interval 0.1 ≤ *f* ≤ 0.9 is recommended as the most relevant for the linear fitting. But by the statement of the problem, we are interested not in these tails but in the vicinity of the zero ordinate of the function stated by Eq. (4). Due to the assumption of the validity of the Hill kinetics, we expect the liner dependence there even if we do not have experimental concentration corresponding to the intermediate interval.

To get the desired linear fit, we take all points with the maximal concentration such that their 0.95 quantile is less than zero, and all points with the minimal concentration such that their 0.05 quantile is larger than zero (and also points, that cross the zero ordinate too). The procedure goes further only if at least two points satisfy this condition. In Fig. 3, there are sets (red and magenta circles highlighted by crosses) corresponding to two concentrations under such conductions, and one set corresponding to three concentrations.

The possibility to use only two concentrations for the linear regression supplied with the uncertainty range is based on the fact that there are several dozen response points for each concentration, i.e. the system has enough degrees of freedom for the uncertainty quantification alongside with a linear fit.

Finally, it should be emphasized that a robust fitting algorithm should be used to eliminate correlation-based effects emerging in the ensemble obtained by the pseudo-replication procedure as mentioned above.

However, we would like to stress that we are interested not in the coefficients of the linear function (4) but in the uncertainty of its zero crossing. It is known [20] that this inverse problem can be solved by the application of the inverse linear fit for the function ln [*x*](*FP*_9_). Although the respective zero crossing point could be shifted due to the interchange of arguments, the uncertainty range of this location is proved to be correct.

***The workflow’s step 3:** To apply the robust polynomial fit of order 1 (it better to use the bisquare weights of points to reduce the influence of large outliers) to the data* ln (*x*) *as a function of FP*_9_*, which belong to the interval between 0.05 and 0.95 quantiles, and compute the prediction interval for the argument* $FP_{9}=0$*, which gives the target uncertainty bounds.*

In Fig. 3, solids lines represents such linear regression lines obtained using the MATLAB’s ’fit’ function to generate the fitting model.

In the examples considered, the default of options of convergence were used: the maximum number of evaluations of the model equal to 600, the maximum number of iterations allowed for the fit equal to 400, and the termination tolerance on both model and coefficient values equal to $10^{-6}$. In all cases simulated, the exit flag reporting that the exit condition of the algorithm indicating convergence within tolerances was achieved for the number of iterations varying from 6 to 34 and their median value equal to 11. The robust bisquare weights fitting, which uses the iteratively reweighted least-squares algorithm, was applied with the default tuning constant equal to 4.685.

Consequently, the prediction interval for the argument $FP_{9}=0$ was calculated using MATLAB’s function ’predint’, which returns upper and lower 95% prediction bounds. The dashed lines highlight such prediction intervals for $-1<FP_{9}<0$; the points of their crossing with the line $FP_{9}=0$ are the target uncertainty bounds.

**Fig. 4:**
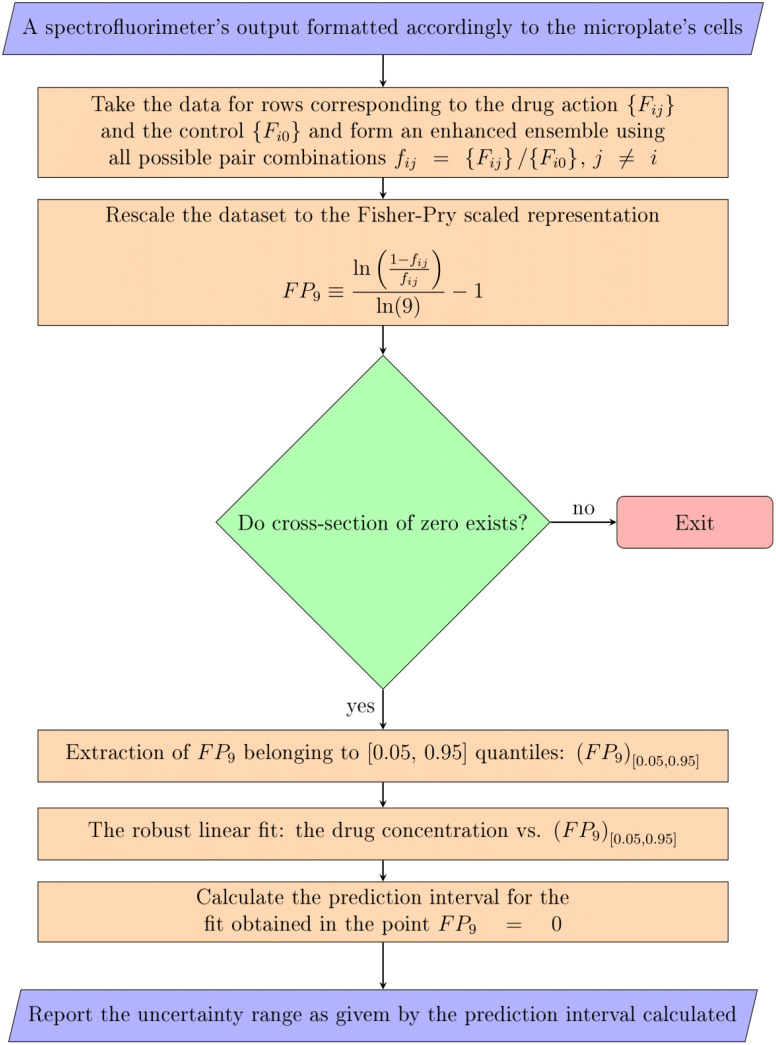
Workflow summarising the algorithm of a single plate-based REMA data processing for obtaining the MIC uncertainty via the pseudo-replication procedure.

***Summary.*** The whole workflow of the proposed approach is summarized in Fig. 4. Two steps of processing above the block of decision rule in this diagram are called ’step1′ and ’step2′ in the text above; the steps below the block of decision rule corresponds to ’step3′ in the text above. In Fig. 4 they are subdivide in subsequent elements to make a better correspondence to the program code provided in Supplementary material. The decision step assures whether these elements leading to the well-posed determination of the uncertainty range can be actually completed. It stops the evaluation when the data do not exhibit indicator response corresponding to the drug action sufficient for the significant growth depression by an applied drug. Simultaneously, it takes into account the cut-off of outliers, which can emerge due to random effects such as spurious chemical indicator response, unintentional random bacterial contamination of some control cell, etc.

Note also that the program implementation described above was carried out by means of MATLAB but the algorithm is not programming language-specific. Its practical realization combines standard functions of statistical analysis and data processing, i.e. the respective program can be easily translated to other widespread programming languages such Python, R, Julia, where the respective statistical packages already exist.

## Method validation

The proposed method described above was applied to 10 antimycobacterial drugs, the list of which is given in Fig. 1 since they represent not only the set of principal medications used for curing tuberculosis but, as seen in the mentioned figure, their minimal inhibitory concentrations vary significantly even for drug-sensitive mycobacteria.

**Fig. 5:**
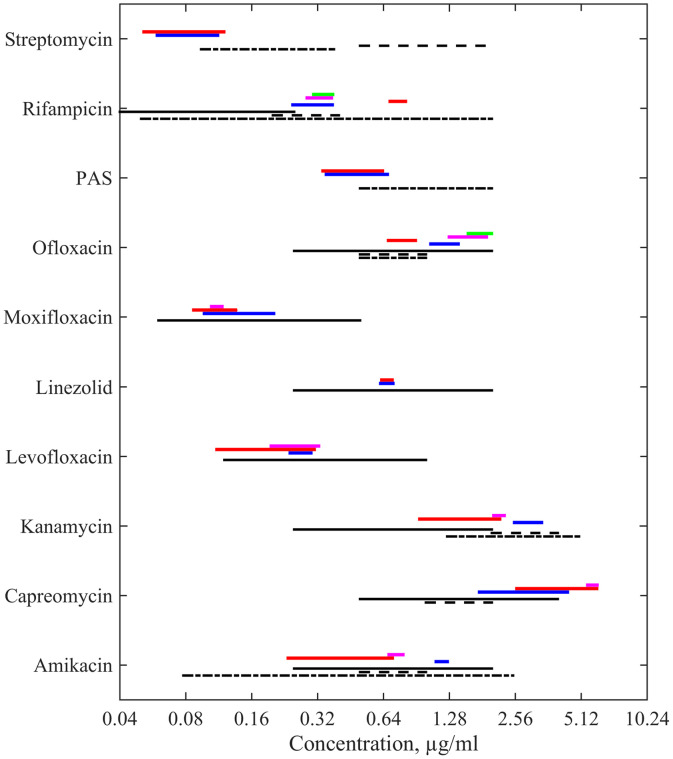
Ranges of uncertainty for 10 investigated antimycobacterial drugs acting on H37Rv strain of *M. tuberculosis* based on the statistical enhancement of data for microplates used in the REMA tests (coloured intervals; separate colours correspond to individual microplates) in comparison with the range reported on the base of statistical processing of multiple independent experiments for the respective MIV determination by different standard methods according to the works [21] (black solid line), [22] (black dashed line), and [7] (black dash-dotted line).

The resulting ranges for the explored standard laboratory strain H37Rv are shown in Fig. 5 as coloured lines. Each colour corresponds to the uncertainty range determined for the fluorescence data obtained from one microbiological plate. One can see that they can not be considered as almost point-wise; sometimes they continue to a considerable extent reaching doubled or even tripled concentrations (the numerical data are provided in Supplementary material). At the same time, such a significant extent assures overlapping of the intervals in the majority of cases (although there are some exceptions of disjoint intervals). Thus, this argues that reporting such intervals obtained from a unique REMA test with one plate (if it is not possible to make several independent tests due to available experimental/clinical conditions) provides more reliable information than one value of the MIC under the same conditions. Simultaneously, collecting such intervals from several repetitions will converge to more substantiated conclusions about a drug’s action.

The obtained ranges can be compared with well-known data compendiums providing the critical meta-analysis of minimal inhibitory concentrations for different antibiotics acting on the strain H37Rv of *M. tuberculosis*. As shown by different black lines in Fig. 5, they also indicate wide ranges of the MIC obtained by processing extensive datasets which includes variability of the MIC determined by visual inspection of the complete growth inhibition in microtiter plates [21] (black solid line), using the Bactec 460-TB radiometric method [22] (black dashed line), and with the fluorescence-based REMA test with the standard averaging over three wells repeated for four microtiter replicates [7] (black dash-dotted line). Our results are coordinated with these wider intervals and, in the majority of cases, correspond to the maxima of the distribution of even wider statistics shown in Fig. 1.

Another advantage of the usage of the enhanced ensemble of data is the fact that the conventional procedure of averaging can lead to a data sequence, which does not follow the well-defined Hill kinetics. This effect can occur not due to the specificity of drug binding but due to the statistical properties of the raw data distribution and is purely artificial. In our data, an example of such a behaviour exhibits the test of para-aminosalicylic acid (PAS).

**Fig. 6:**
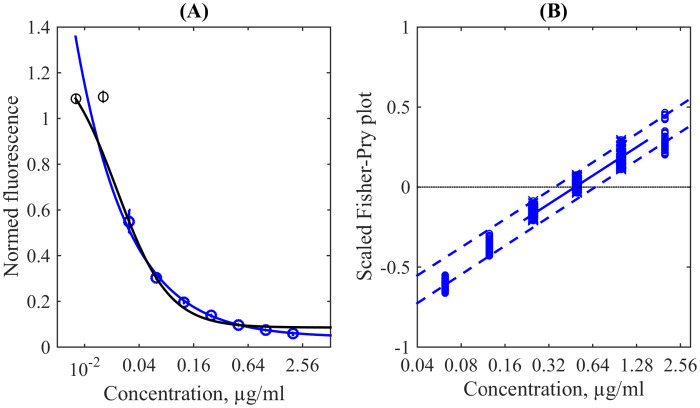
(A) The conventional representation of data for the REMA test with PAS obtained by the preliminary averaging over wells (circles, where the black ones denote values larger than one) and two Hill’s curves fitting their full set (black) and only those, which values are less than one (blue). (B) The Fisher-Pry plot according to Eq. (4) for the enhanced ensemble (markers); the solid line is the linear fit in the vicinity of zero crossing and the dashed lines are upper and lower 95% prediction bounds. (For interpretation of the references to colour in this figure legend, the reader is referred to the web version of this article).

Fig. 6 (A) demonstrates the dependence of the relative fluorescence on the PAS concentration obtained by the conventional averaging over the plate’s columns shown as circle markers with error bars (the latter are almost everywhere shorted than the radius of a circle, i.e. the formal uncertainty is small). Note that the two first data points (black circles) have values larger than one and slightly growing with the subinhibitory growth of concentration. Such an effect has a character of hormesis [23], [24]. In this case, the usage of the four-parametric Eq. (1) leads to questionable results. When the data points *f* < 1 are used, the fit is quite accurate respectively to them but gives completely unreliable large *f*_max_, see the blue curve in Fig. 6 (A). When all data points are used, the fitting curve looks more reasonably sigmoidal but it significantly deviates from the actual data, see the black curve in Fig. 6 (A).

On the contrary, the usage of the scaled Fisher-Pry representation given by Eq. (4) applied to the enhanced ensemble of original data results in the stripe of points well-bounded by the straight lines, see Fig. 6 (A). Note that Eq. (4) operates with the exact asymptotics of the kinetic dependence equal to $f_{\min}=0$ and $f_{\max}=1$, i.e. linearity of the stripe in this representation argues in favour of the biophysically-correct Hill’s pharmacokinetics. The linear fit in the vicinity of the zero crossing is shown as the solid straight line and its 95% uncertainty prediction range (dashed straight lines) bounds practically all data points directly obtained from the total ensemble of measurements in all wells of the test plate. Therefore, we can consider the interval between the points of crossing of the dashed lines with the horizontal line $FP_{9}=0$ as the MIC uncertainty range [0.35, 0.67] *μ*g/ml, which can be established in this experiment. Its reduction to one MIC value does not satisfy the statistical scattering of the actual data. Moreover, Fig. 6 (B) explains that these scattered data satisfy the standard biochemical kinetics, but the contradictions seen in Fig. 6 (A) originated from improper simplifications in averaging without taking into account such uncertainty.

## Limitations

Accuracy of the MIC bounds determination depends on the number of concentrations resulting in the data points of *FP*_9_, which belong to the interval between 0.05 and 0.95 quantiles. Although formally of can get a unique straight line going through one point with *FP*_9_ > 0 and one point with *FP*_9_ > 0, the more reliable predictions require at least one additional point with the intermediate response. In addition, one should be sure that the row containing the control wells should be well-calibrated since the records of its fluorescence are used to normalize the data. The method is also limited by the drugs, which do lead to the strong effect of hormesis. This situation leads to multiple zero-crossing. In principle, they can be automatically classified as leading to growth-depressing and stimulating effects by the pairwise comparison but this is left for future works. But in practice, one can exclude rows demonstrating the effect of hormesis from the input table via in the hand-mode.

Note also that we considered only the case of *M. tuberculosis*, for which a variety of reliable reference data is reported in the literature respectively to the accepted first- and second-line drugs that made possible the validation of the proposed method.

At the same time, recent efforts aimed at identifying new drug candidates capable of combating emerging drug resistance increasingly rely on the use of non-pathogenic Mycobacterium species as surrogate models. These models are more accessible to general laboratories that are not accredited to work with hazardous pathogens [25].

Among such species, one can list *M. smegmatis* [26], [27], the number of data for which counts during the last years. Thus, we can denote the tests of the proposed data interpretation model for the minimal inhibitory concentration tests for this bacterium as a promising goal for future studies.

## Ethics statements

No experiments with humans and animals were conducted for this study.

## CRediT authorship contribution statement

**Eugene B. Postnikov:** Conceptualization, Methodology, Investigation, Software, Visualization, Writing – original draft, Writing – review & editing. **Anastasia I. Lavrova:** Conceptualization, Data curation, Investigation, Resources, Writing – review & editing, Project administration.

## Declaration of competing interest

The authors declare that they have no known competing financial interests or personal relationships that could have appeared to influence the work reported in this paper.

## Data Availability

URL of the data repositoty is provided in the text
