# Supplementary material for: Quantifying uncertainty of tuberculosis drug susceptibility range from single-microplate test
Source: MethodsX. 2026 Jan 14;16:103797. doi: 10.1016/j.mex.2026.103797 (PMC12854056; doi:10.1016/j.mex.2026.103797)
Supplement: Supplementary file 1 [file mmc1.pdf]

Supplementary Material

Quantifying uncertainty of tuberculosis drug susceptibility range from single-microplate test

Eugene B. Postnikov[1] and Anastasia I. Lavrova[2,3]

[1] Department of Theoretical Physics, Kursk State University, Radishcheva st., 33, Kursk 305000, Russia

[2] Sophya Kovalevskaya North-West Mathematical Research Center,, Immanuel Kant Baltic Federal University, Nevskogo St. 14, Kaliningrad 236041, Russia

[3] Saint-Petersburg State Research Institute of Phthisiopulmonology, Ligovskiy Prospect 2-4, 194064 Saint Petersburg, Russia

Fig. S1: plots of the median fluorescence intensity f (markers) normed to the same of the control as a function of the drug concentration C for different drugs the regression of each individual data set (lines) based on the Hill kinetics (the explicit form of the equations and its parameters are given below in Table S1).

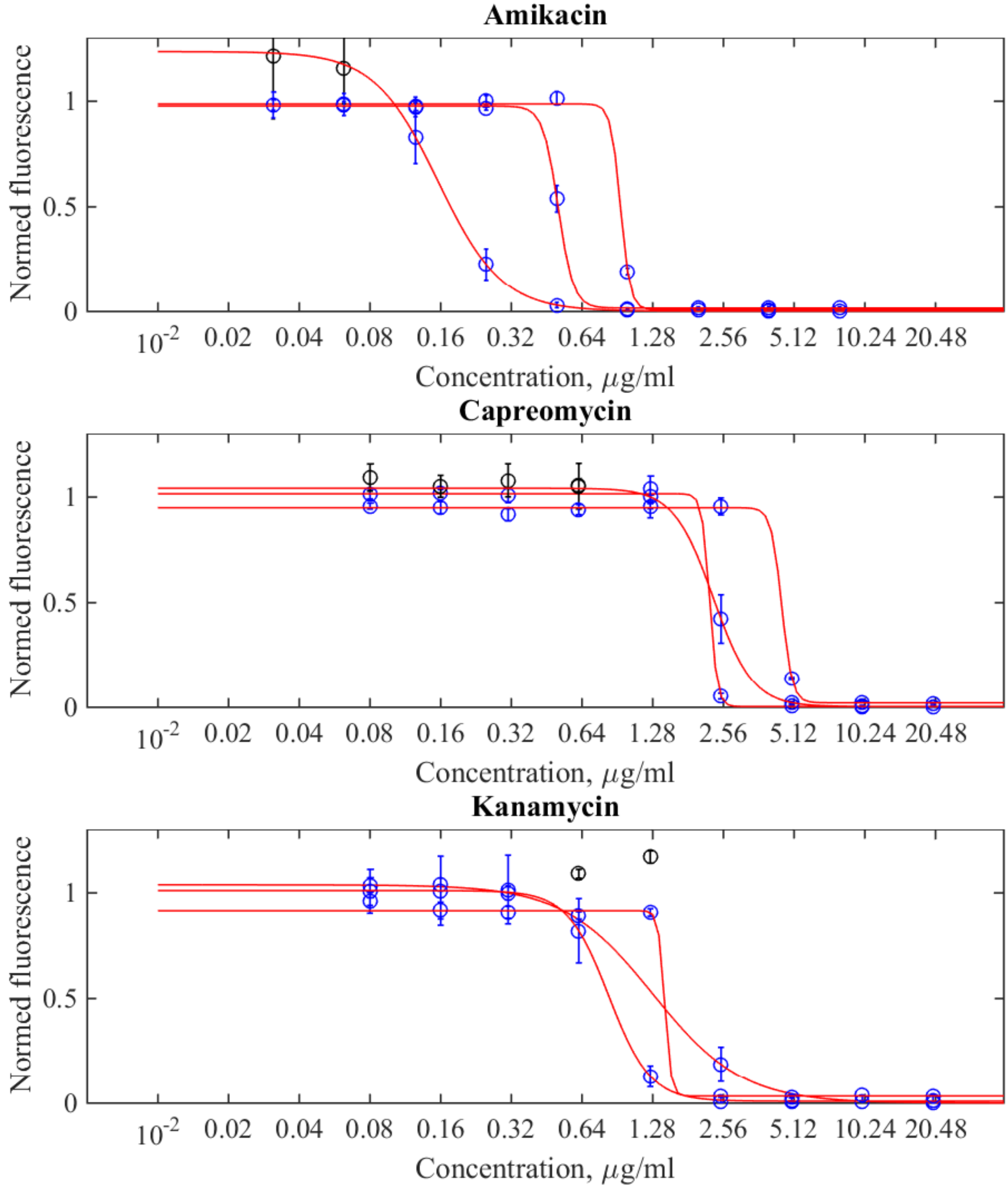

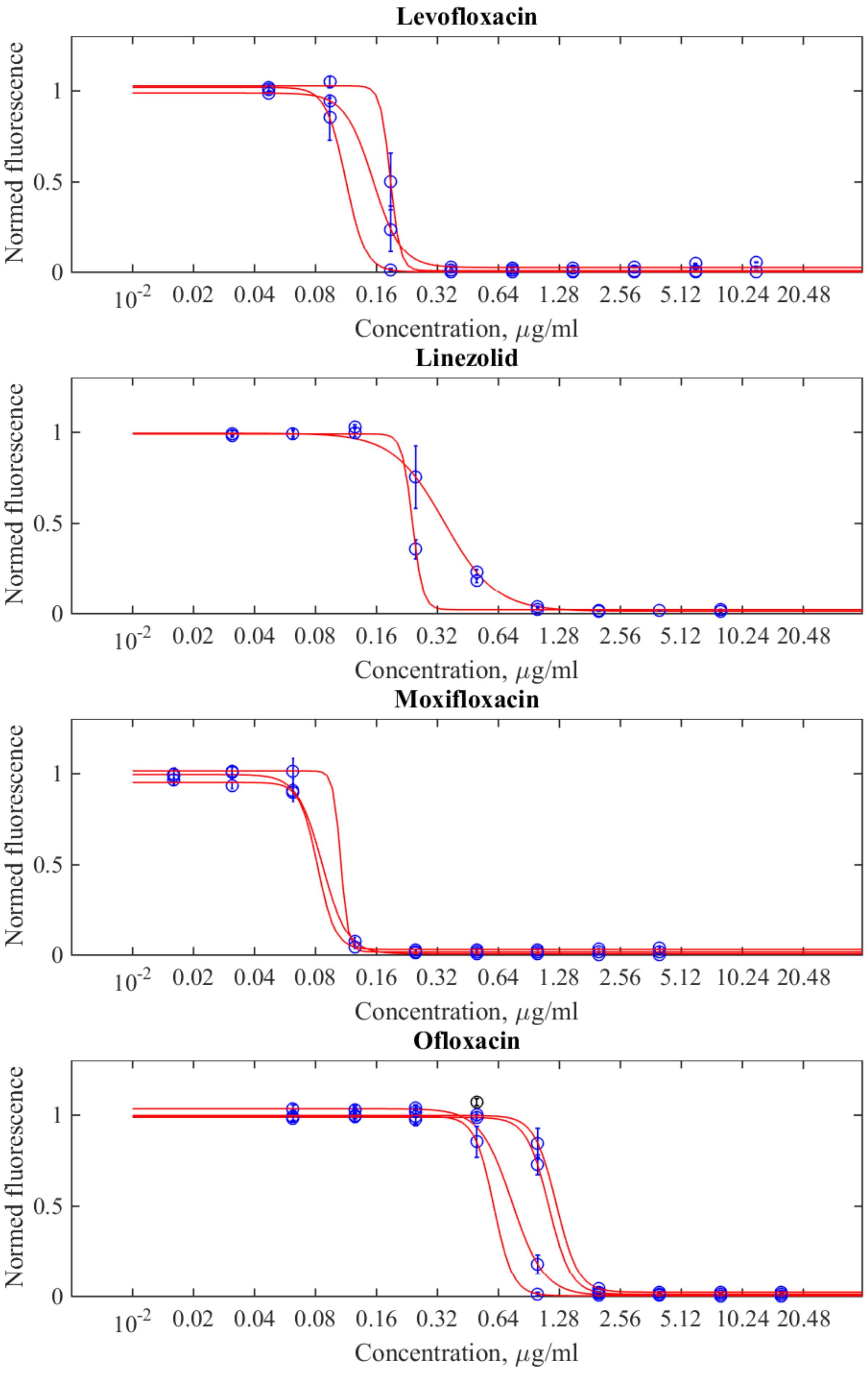
Levofloxacin
Linezolid
Moxifloxacin
Ofloxacin
Normed fluorescence
0
0.5
1
10^{-2} 0.02 0.04 0.08 0.16 0.32 0.64 1.28 2.56 5.12 10.24 20.48
Concentration, μg/ml

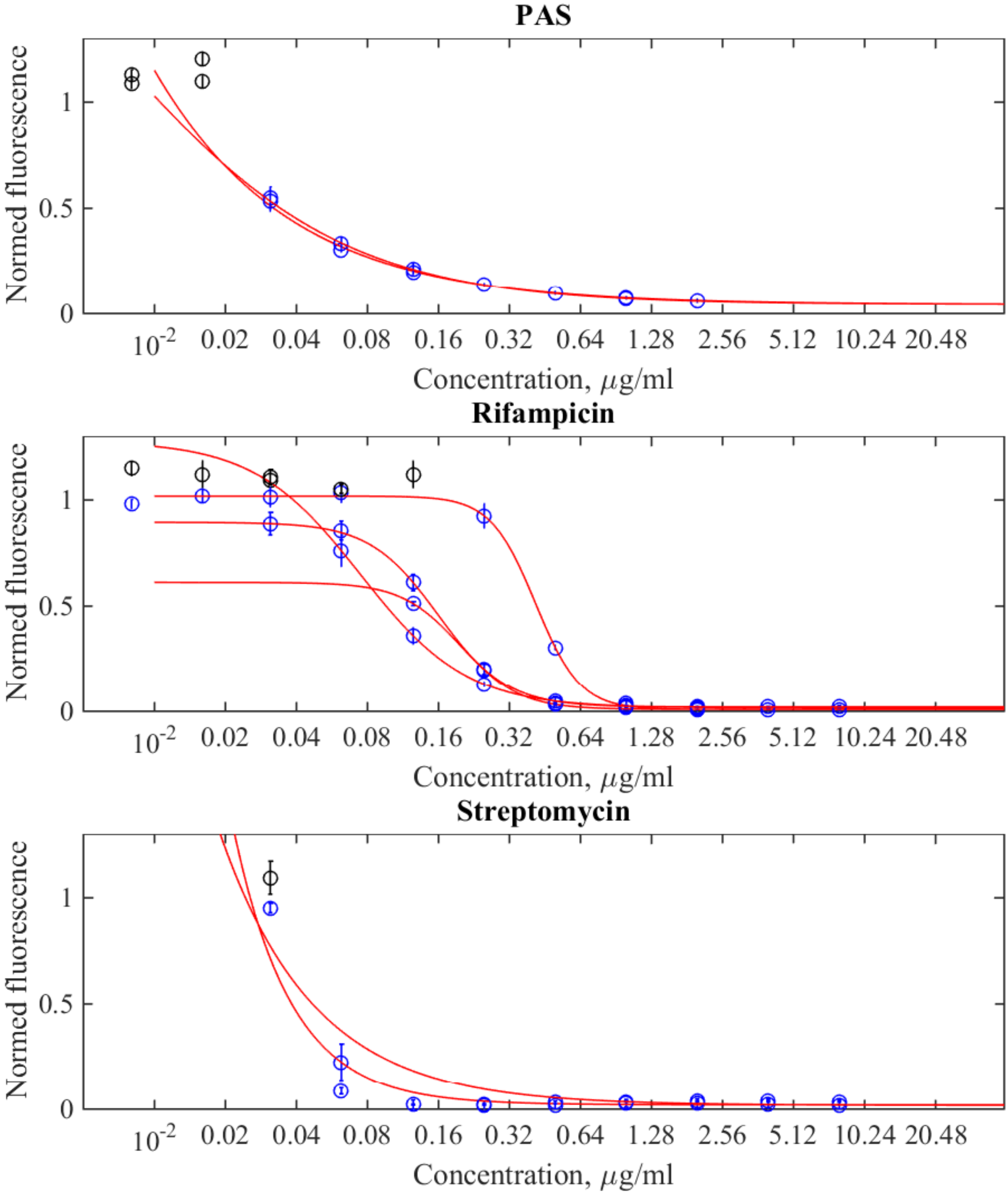
PAS
Normed fluorescence
0
0.5
1
10^{-2}
0.02
0.04
0.08
0.16
0.32
0.64
1.28
2.56
5.12
10.24
20.48
Concentration, μg/ml
Rifampicin
Normed fluorescence
0
0.5
1
10^{-2}
0.02
0.04
0.08
0.16
0.32
0.64
1.28
2.56
5.12
10.24
20.48
Concentration, μg/ml
Streptomycin
Normed fluorescence
0
0.5
1
10^{-2}
0.02
0.04
0.08
0.16
0.32
0.64
1.28
2.56
5.12
10.24
20.48
Concentration, μg/ml

Table S1: Parameters of the Hill function describing the median fluorescence intensity f normed to the same of the control

$$f(C) = f_{min} + \frac{f_{max} - f_{min}}{1 + \left(\frac{C}{IC50}\right)^{\alpha}}$$

as a function of the drug concentration C, supplied with their 95% confidence bounds (CI).

No	Name	f_{min}	$CI_{f_{min}}$	f_{max}	$CI_{f_{max}}$	$IC50$	CI_{IC50}	α	CI_{α}
1	Amikacin	0.0085	(-0.0151,0.032)	0.99	(0.967,1.01)	0.93	(-111,113)	21	(-3.68e+04,3.68e+04)
2	Amikacin	0.0012	(-0.00619,0.00866)	1.2	(0.811,1.66)	0.16	(0.115,0.197)	3.2	(2.3,4.09)
3	Amikacin	0.016	(0.00652,0.026)	0.98	(0.968,0.986)	0.51	(0.396,0.617)	13	(-214,241)
4	Capreomycin	0.0034	(-0.0166,0.0234)	1	(0.995,1.04)	2.2	(-3.19e+03,3.19e+03)	27	(-3.45e+05,3.46e+05)
5	Capreomycin	0.0033	(-0.0169,0.0234)	1	(0.954,1.13)	2.3	(2.16,2.47)	5.2	(2.48,7.85)
6	Capreomycin	0.021	(-0.00858,0.0515)	0.95	(0.931,0.969)	4.5	(-181,190)	20	(-8.17e+03,8.21e+03)
7	Kanamycin	3.9e-14	(NaN,NaN)	1	(0.995,1.08)	1.3	(0.914,1.66)	2.3	(1.47,3.1)
8	Kanamycin	0.0095	(0.000754,0.0182)	1	(1,1.02)	0.83	(0.815,0.851)	4.9	(4.61,5.22)
9	Kanamycin	0.034	(0.000544,0.0668)	0.92	(0.887,0.944)	1.4	(-4.39e+04,4.39e+04)	33	(-7e+06,7e+06)
10	Levofloxacin	0.0082	(-0.00668,0.023)	1	(0.999,1.06)	0.19	(-0.148,0.522)	18	(-9.49e+03,9.53e+03)
11	Levofloxacin	0.002	(0.000549,0.00343)	1	(1.02,1.02)	0.11	(0.112,0.114)	8.9	(8.4,9.44)
12	Levofloxacin	0.026	(0.0145,0.0375)	0.99	(0.959,1.02)	0.15	(0.146,0.162)	6.2	(4.84,7.65)
13	Linezolid	0.013	(-0.0149,0.0405)	0.99	(0.962,1.03)	0.35	(0.322,0.372)	3.4	(2.8,4.06)
14	Linezolid	0.021	(-0.039,0.0818)	0.99	(0.898,1.08)	0.24	(-8.96,9.44)	17	(-1.77e+04,1.77e+04)
15	Moxifloxacin	0.007	(-0.00097,0.0149)	1	(0.983,1.01)	0.086	(0.0839,0.089)	7.1	(6.5,7.64)
16	Moxifloxacin	0.017	(0.0109,0.0229)	1	(1.01,1.02)	0.11	(-5.09,5.3)	23	(-7.14e+03,7.19e+03)
17	Moxifloxacin	0.031	(0.0176,0.0434)	0.95	(0.932,0.972)	0.082	(0.0734,0.0905)	10	(6.44,13.6)
18	Ofloxacin	0.0055	(-0.00321,0.0142)	1	(1.02,1.05)	0.75	(0.589,0.907)	5.5	(1.44,9.53)
19	Ofloxacin	0.0034	(-0.0108,0.0177)	0.99	(0.977,1.01)	0.61	(0.546,0.67)	9.2	(4.52,13.9)
20	Ofloxacin	0.012	(-0.00136,0.025)	0.99	(0.977,1)	1.1	(1.07,1.2)	8	(4.37,11.6)
21	Ofloxacin	0.023	(0.00879,0.0378)	1	(0.986,1.01)	1.2	(1.16,1.31)	8	(5.9,10.1)
22	PAS	0.042	(-0.12,0.204)	41	(-1.21e+04,1.22e+04)	9.6e-05	(-0.0388,0.039)	0.77	(-1.31,2.85)
23	PAS	0.043	(0.0275,0.0582)	2.4	(-0.748,5.65)	0.0066	(-0.00807,0.0213)	0.88	(0.668,1.09)
24	Rifampicin	0.017	(0.0131,0.0203)	1.3	(1.19,1.37)	0.075	(0.0688,0.0809)	1.9	(1.82,2.02)
25	Rifampicin	0.0088	(-0.0363,0.054)	1	(0.989,1.05)	0.41	(0.383,0.442)	4.6	(3.39,5.75)
26	Rifampicin	0.0091	(0.000228,0.018)	0.61	(0.486,0.738)	0.2	(0.163,0.235)	3.5	(2.15,4.77)
27	Rifampicin	0.021	(0.0117,0.0293)	0.89	(0.878,0.912)	0.16	(0.154,0.165)	3	(2.75,3.23)
28	Streptomycin	0.02	(-0.0363,0.0769)	2.9e+02	(-5.15e+06,5.15e+06)	0.001	(-10.5,10.5)	1.8	(-10.8,14.4)
29	Streptomycin	0.018	(-0.281,0.316)	7.1e+02	(-6.3e+06,6.3e+06)	5.7e-05	(-0.471,0.471)	1.1	(-5.42,7.59)

Table S2: Uncertianty ranges for the minimal inhibitory concentrations (MIC) of drugs acting on the strain h37Rv of *M. tuberculosis* determined by the proposed method for the processing of the enhanced ensemble of fluorescence-based data obteined in the resazurin microtiter assay (REMA).

No	Name	MIC range (μg/ml)
1	Amikacin	[1.1,1.3]
2	Amikacin	[0.23,0.7]
3	Amikacin	[0.68,0.78]
4	Capreomycin	[1.8,4.4]
5	Capreomycin	[2.6,6]
6	Capreomycin	[5.5,6]
7	Kanamycin	[2.5,3.4]
8	Kanamycin	[0.94,2.2]
9	Kanamycin	[2,2.3]
10	Levofloxacin	[0.24,0.3]
11	Levofloxacin	[0.11,0.31]
12	Levofloxacin	[0.2,0.32]
13	Linezolid	[0.62,0.71]
14	Linezolid	[0.63,0.7]
15	Moxifloxacin	[0.097,0.2]
16	Moxifloxacin	[0.087,0.14]
17	Moxifloxacin	[0.1,0.12]
18	Ofloxacin	[1.1,1.4]
19	Ofloxacin	[0.67,0.9]
20	Ofloxacin	[1.3,1.9]
21	Ofloxacin	[1.6,2]
22	PAS	[0.35,0.67]
23	PAS	[0.34,0.63]
24	Rifampicin	[0.25,0.37]
25	Rifampicin	[0.69,0.81]
26	Rifampicin	[0.29,0.37]
27	Rifampicin	[0.31,0.37]
28	Streptomycin	[0.059,0.11]
29	Streptomycin	[0.052,0.12]
